# Targeting SHP-1-STAT3 signaling: A promising therapeutic approach for the treatment of cholangiocarcinoma

**DOI:** 10.18632/oncotarget.17779

**Published:** 2017-05-10

**Authors:** Ming-Hung Hu, Li-Ju Chen, Yen-Lin Chen, Ming-Shen Tsai, Chung-Wai Shiau, Tzu-I Chao, Chun-Yu Liu, Jia-Horng Kao, Kuen-Feng Chen

**Affiliations:** ^1^ Department of Medical Research, National Taiwan University Hospital, Taipei, Taiwan; ^2^ National Taiwan University College of Medicine, Taipei, Taiwan; ^3^ Graduate Institute of Clinical Medicine, College of Medicine, National Taiwan University, Taipei, Taiwan; ^4^ Division of Hematology and Oncology, Department of Medicine, Cardinal Tien Hospital, New Taipei City, Taiwan; ^5^ School of Medicine, Fu Jen Catholic University, New Taipei City, Taiwan; ^6^ Department of Oncology, Taipei Veterans General Hospital, Taipei City, Taiwan; ^7^ Department of Pathology, Cardinal Tien Hospital, New Taipei City, Taiwan; ^8^ Institute of Biopharmaceutical Sciences, National Yang-Ming University, Taipei, Taiwan; ^9^ Transplant Medicine and Surgery Research Centre, Changhua Christian Hospital, Changhua, Taiwan; ^10^ School of Medicine, National Yang-Ming University, Taipei, Taiwan; ^11^ Division of Gastroenterology and Hepatology, Department of Internal Medicine, National Taiwan University Hospital, Taipei, Taiwan

**Keywords:** cholangiocarcinoma, SC-43, STAT3, SHP-1, inflammatory cancer

## Abstract

Sorafenib is a multiple kinase inhibitor which targets Raf kinases, VEGFR, and PDGFR and is approved for the treatment of hepatocellular carcinoma (HCC). Previously, we found that p-STAT3 is a major target of SC-43, a sorafenib derivative. In this study, we report that SC-43-induced apoptosis in cholangiocarcinoma (CCA) via a novel mechanism. Three CCA cell lines (HuCCT-1, KKU-100 and CGCCA) were treated with SC-43 to determine their sensitivity to SC-43-induced cell death and apoptosis. We found that SC-43 activated SH2 domain-containing phosphatase 1 (SHP-1) activity, leading to p-STAT3 and downstream cyclin B1 and Cdc2 downregulation, which induced G2-M arrest and apoptotic cell death. Importantly, SC-43 augmented SHP-1 activity by direct binding to N-SH2 and relief of its autoinhibition. Deletion of the N-SH2 domain (dN1) or point mutation (D61A) of SHP-1 counteracted the effect of SC-43-induced SHP-1 phosphatase activation and antiproliferation ability in CCA cells. In vivo assay revealed that SC-43 exhibited xenograft tumor growth inhibition, p-STAT3 reduction and SHP-1 activity elevation. In conclusion, SC-43 induced apoptosis in CCA cells through the SHP-1/STAT3 signaling pathway.

## INTRODUCTION

Cholangiocarcinoma (CCA) is the second most common hepatic malignancy after hepatocellular carcinoma (HCC) and the most common biliary malignancy, accounting for 3% of all gastrointestinal tumors [1]. CCAs are epithelial malignancies that arise from cholangiocytes and are characterized by aggressive behavior and advanced clinical stage. Although CCA is not a common malignancy, the overall incidence of CCA has increased significantly over the past three decades [2–4]. Meanwhile, the mortality from CCA is still increasing [4]. Currently, not a single targeted agent has been approved for the treatment of CCA. Treatment options for CCA are limited, and cytotoxic chemotherapy remains the only choice for unresectable metastatic CCA. However, CCA is generally refractory to most chemotherapy, and 5-year survival rate is extremely poor, remaining at 10% [5, 6]. The unmet medical need in patients with CCA remains a great clinical challenge. Owing to the unsatisfactory therapeutic outcomes under the current standard management, development of novel agents for treatment of CCA is undoubtedly an important mission.

CCA is an endemic disease, the worldwide incidence of which varies greatly [7, 8]. Incidence in regions such as Thailand in Southeast Asia (as high as 113 per 100,000 in men and 50 per 100,000 in women) is about one hundred times more than that in most Western countries. There are several well-established risks associated with CCA development, including infestation with the liver fluke *Opisthorchis viverrini,* hepatolithiasis, primary sclerosing cholangitis (PSC), choledochal cysts, liver cirrhosis, alcohol consumption, tobacco use, and chronic viral hepatitis [6, 9, 10]. Generally speaking, chronic inflammation significantly contributes to CCA formation. According to epidemiologic and population-based studies, CCA incidence is still increasing in Thailand and is strongly correlated with the high prevalence of infection with the parasite *O.viverrini* [7, 11]. These studies have provided clues to the role of environmental factors in the etiology and pathogenesis of cholangiocarcinoma. *O.viverrini* infection represents a classical model for CCA that interprets the role of inflammation in CCA carcinogenesis well [12, 13].

Cancer-associated inflammation is marked by the presence of specific inflammatory cells and inflammatory mediators, including cytokines and chemokines.

Signal transducers and activators of transcription 3 (STAT3) belong to a family of transcription factors that relay cytokine receptor-generated signals into the nucleus. STAT3 is activated by the cytokine IL-6 as well as other growth factors, including epidermal growth factor receptor (EGFR), fibroblast growth factor receptor (FGFR), and platelet-derived growth factor receptor (PDGFR) through tyrosine phosphorylation [14]. After dimerization, STAT3 translocates into the nucleus where it activates gene transcription. STAT3 signaling mediates cell growth, proliferation, inflammatory cytokine production, cell invasion and migration. Stimulations such as *O.viverrini* infection or PSC cause cholestasis and chronic inflammation of the bile duct, which can induce a variety of cytokines including IL-6, platelet-derived growth factor (PDGF), and epidermal growth factor (EGF) [15, 16]. This inflammatory cascade activates STAT3, leading to overproduction of bile duct epithelium growth factor, thus promoting CCA initiation. Because of the role of STAT3 in inflammation and cancer development, targeting STAT3 is a rational treatment strategy for CCA.

Sorafenib acts as a multiple kinase inhibitor that works against rapidly accelerated fibrosarcoma (Raf) kinases, vascular endothelial growth factor receptor (VEGFR), and PDGFR, among others. Boris et al. revealed that sorefenib inhibits CCA cells by downregulating STAT3 signaling [17]. Previously, we discovered that SHP-1, a nonreceptor protein tyrosine phosphatase (PTP) that negatively regulates p-STAT3, is also a direct target of sorafenib [18, 19]. Accordingly, we have synthesized a series of sorafenib analogs which resemble sorafenib structure closely but have no kinase inhibition activities. Among these derivatives, SC-43 was found to be a more potent SHP-1 agonist than sorafenib. Our earlier study demonstrated that SC-43 had therapeutic potential in HCC treatment [18]. Based on this preclinical success, SC-43 is currently poised to enter a phase I clinical trial for treatment of HCC.

Given the evidence for the antiproliferative ability of SC-43 in CCA through STAT3 inhibition, we hypothesize that it might have a therapeutic effect in CCA. In the present study, we assessed the effect of SC-43 on CCA cells and investigated the underlying molecular mechanism.

## RESULTS

### Novel sorafenib derivative SC-43 induced apoptosis in CCA cells by inducing G2-M arrest

**Figure 1:**
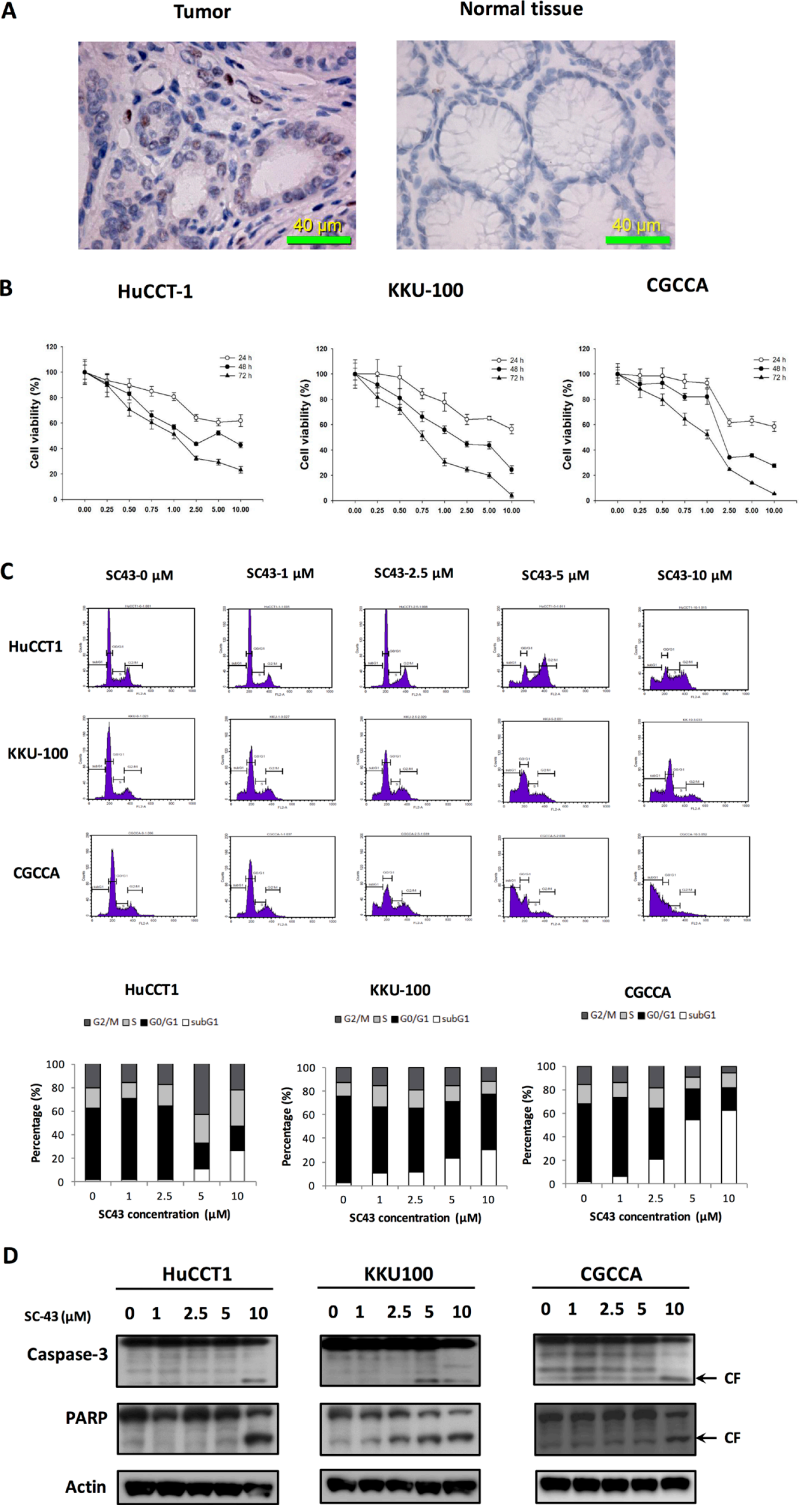
SC-43 exerts anti-proliferative and apoptosis-inducing effects in cholangiocarcinoma (CCA) cells SC-43, a derivative of sorafenib induces apoptosis in CCA cell lines. (**A**) immunohistochemical (IHC) staining for p-STAT3 in CCA tumor (left), compared with normal tissue part (right). (**B**) dose-escalation effects of SC-43 on cell viability. Cells were exposed to SC-43 at the indicated doses for 24, 48, and 72 hours respectively and cell viability was assessed by MTT assay. Points, mean; bars, SD (*n =* 9). (**C**) effects of SC-43 on apoptosis. Cells were exposed to SC-43 at the indicated doses for 24 hours and number of apoptotic cells was determined by flow cytometry. Columns, mean; bars, SD (*n =* 3). (**D**) SC-43-induced apoptosis-related signals in cells.

In CCA cells from representative tumor tissue from a CCA patient, p-STAT3 showed positive expression in the tumor part (Figure 1A, left) compared with normal tissue part (Figure 1A, right). SC-43 is a novel derivative of sorafenib. To investigate the apoptosis effect induced by SC-43, we tested three CCA cell lines: HuCCT-1, KKU-100, and CGCCA. First, as shown in Figure 1B, MTT assay revealed the anti-proliferative effects of SC-43 in CCA cell lines in a dose-dependent manner after treating 24, 48 and 72 hours respectively. Next, flow cytometry analysis showed increased sub-G1 cells and G2-M arrest, indicating SC-43 induced differential apoptotic effects in these cell lines, which corresponds with the MTT assay (Figure 1C). In addition, CCA cells treated with SC-43 demonstrated significant increase in cleaved caspase-3 and PARP level in western blot analysis after exposure for 24 hours (Figure 1D). Taken together, these data indicated that SC-43 has a significant effect to induce G2-M arrest,on CCA cell, leading to apoptosis and growth inhibition.

### SC-43 induces apoptosis with downregulation of STAT3 in CCA cells

**Figure 2:**
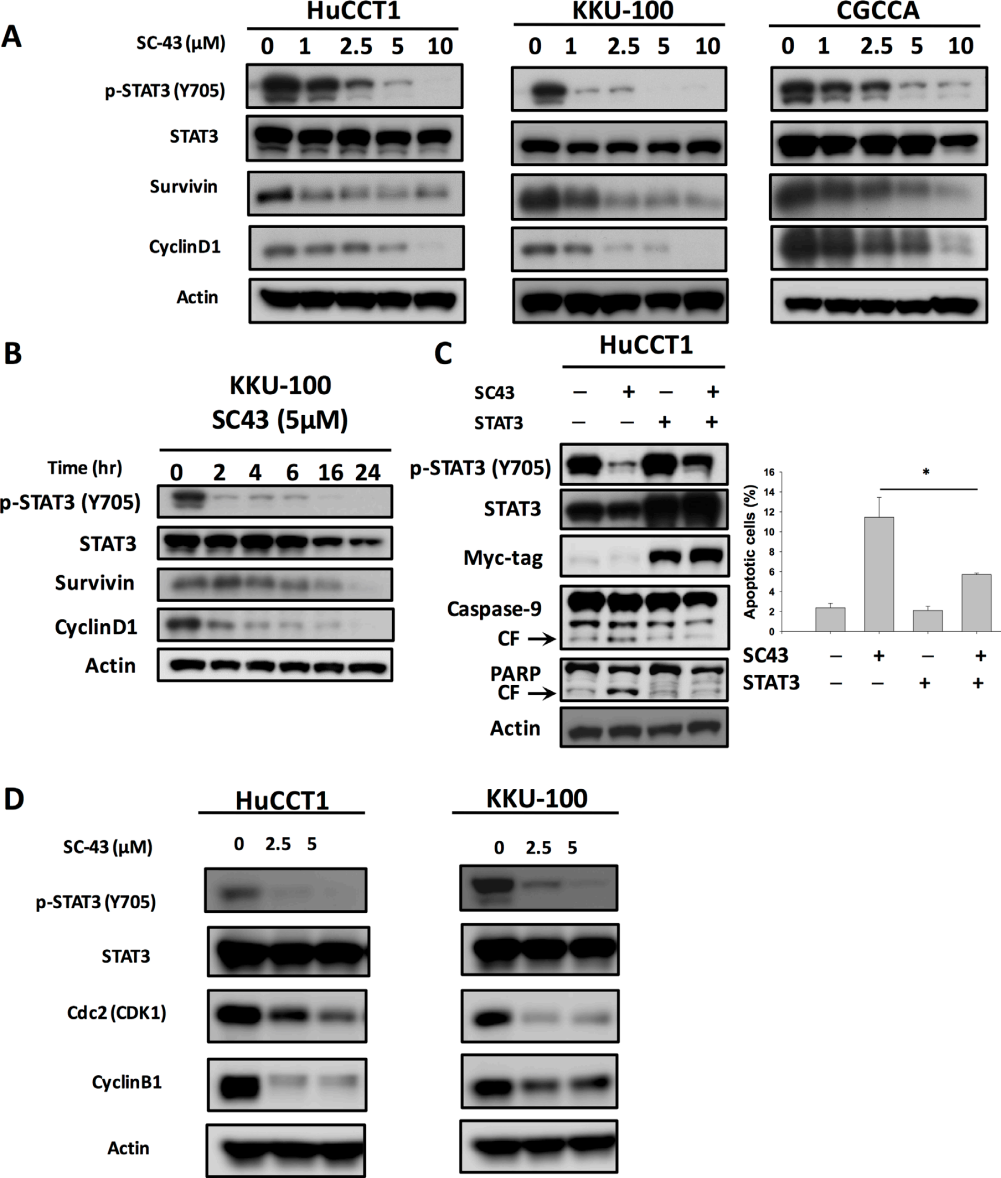
Inhibition of p-STAT3 determines the sensitizing effects of SC-43 in CCA cells (**A**) dose-dependent effects of SC-43 on STAT3-related proteins. Cell were treated with SC-43 at the indicated doses for 24 hours.(**B**) SC-43 induced p-STAT3 inactivation in a time-dependent manner. (**C**) STAT3 reverses the apoptotic effect of SC-43. HuCCT-1 cells transiently expressing STAT3 with Myc-tag were treated with SC-43 at 5 μM for 24 hours and the percentage of apoptosis was measured by sub-G1 analysis. (**D**) western blot analysis of the cyclin B1 and Cdk1 (Cdc2) with SC-43 at the indicated doses for 24 hours. Columns, mean; bars, SD (*n =* 3); *P < 0.05.

Next, we examined whether STAT3 had a relationship with SC-43-induced apoptosis in CCA cells. In Figure 2A, SC-43 was demonstrated to dose-dependently downregulate p-STAT3 and its downstream mediators, survivin and cyclin D-1. STAT3 level did not diminish after SC-43 treatment. Furthermore, SC-43-induced downregulation of the p-STAT3 signaling pathway in KKU-100 was time-dependent (Figure 2B), as well as in HuCCT-1 and CGCCA (Supplementary Figure 1). In STAT3 overexpressing cholangiocarcinoma cells, inhibition of p-STAT3 after SC-43 treatment was reduced. The apoptosis in STAT3 overexpressing cancer cells was reversed as well (Figure 2C). We further explored the role of SC-43 involving G2-M arrest. We performed western blot analysis of cell extracts to compare the levels of expression of the cyclin B1 and Cdk1 (Cdc2), the major regulatory protein and kinase for progression from G2 to M phase. Western blotting demonstrated that the levels of the cyclin B1 and Cdc2 protein were markedly reduced after SC-43 treatment (Figure 2D). In summary, we hypothesized that SC-43 had an antiproliferative effect on CCA cells through inhibiting the STAT3 pathway, as well as G2-M arrest by inhibiting cyclin B1 and Cdc2

### Validation of the SHP-1/p-STAT3 pathway as a molecular determinant of SC-43 induced CCA cell apoptosis

**Figure 3:**
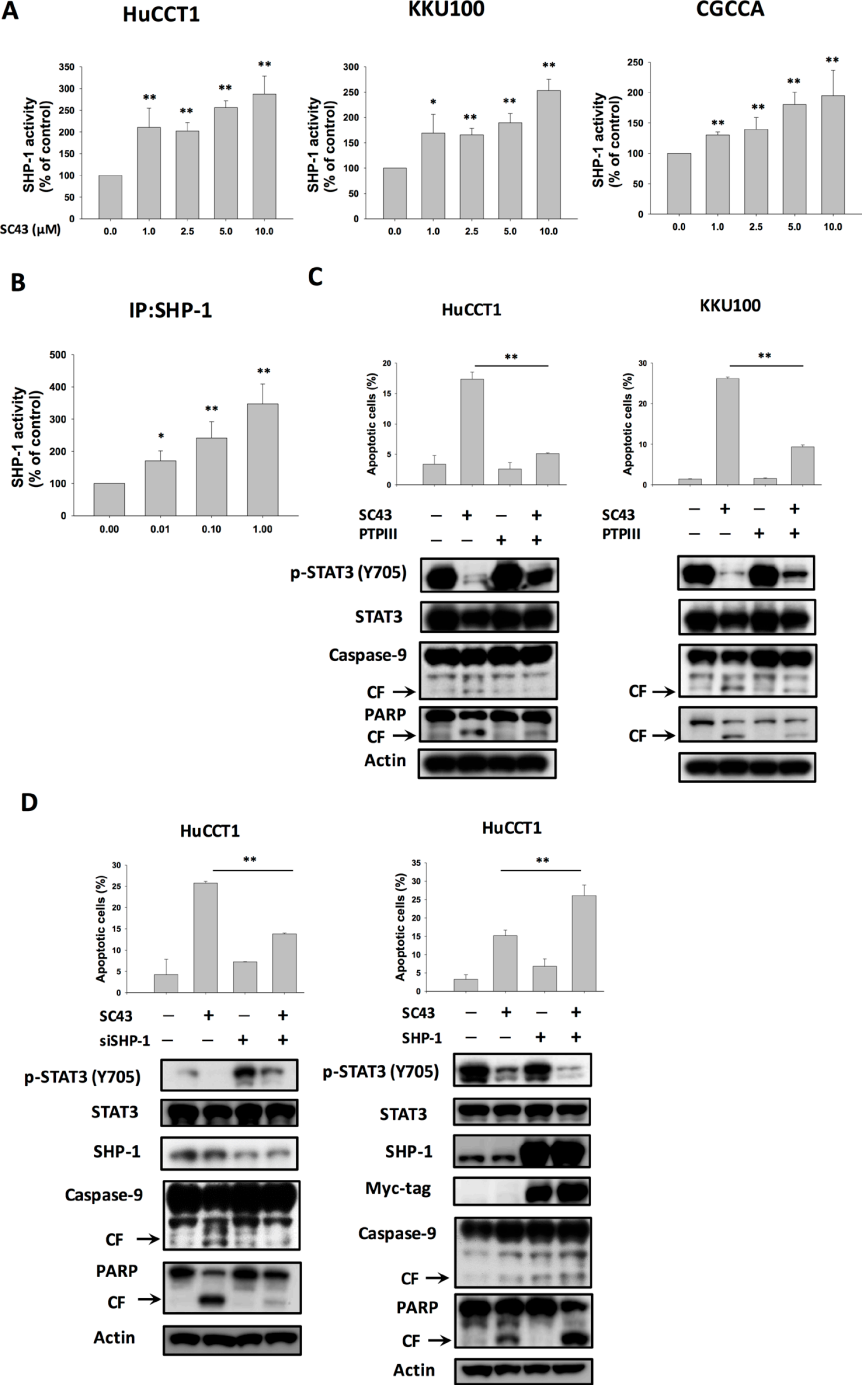
SHP-1/p-STAT3 mediates SC-43-induced apoptosis in CCA cells SHP-1 plays a role in SC-43-induced STAT3 inhibition and apoptosis. (**A**) SC-43 increased SHP-1 activity in CCA cells. Cells were treated with SC-43 at the indicated doses for 16 hours. Columns, mean; bars, SD (*n =* 3). *P < 0.05; **P < 0.01. (**B**) SC-43 activates SHP-1 directly. SC-43 increases the phosphatase activity of SHP-1 in IP-SHP-1 cell lysate from HuCCT-1 cells. Columns, mean; bars, SD (*n =* 3). *P < 0.05; **P < 0.01. (**C**) Protective effects of SHP-1 specific inhibitor (PTPIII) on SC-43-induced apoptosis in HuCCT-1 and KKU-100 cells. Cells were pretreated with PTPIII at 100 nM for 30 minutes before SC-43 treatment. (**D**) left, knockdown of SHP-1 reduces the effects of SC-43 on p-STAT3 and apoptosis. HuCCT-1 cells were transfected with control siRNA or SHP-1 siRNA for 24 hours and then treated with SC-43 for another 24 hours. Right, overexpression of SHP-1 induces more apoptosis with SC-43. Apoptotic assay was performed by sub-G1 analysis. Columns, mean; bars, SD (*n =* 3). **P < 0.01.

In our previous study, we found that SC-43 induced cancer cell apoptosis through upregulation of SHP-1 and downregulation of p-STAT3, suggesting that SHP-1 is a target of SC-43 [18]. Therefore, in this study, we assumed that SHP-1 may also play a role in the biological reaction associated with apoptosis induced by SC-43 in CCA cells. First, we tested SHP-1 phosphatase activity in the above three CCA cell lines: HuCCT-1, KKU-100, and CGCCA. As illustrated in Figure 3A, SHP-1 activity was universally increased in all cell lines after SC-43 treatment. Furthermore, SC-43 increased the phosphatase activity of SHP-1 in IP-SHP-1 cell lysate from HuCCT-1 cells, suggesting that SC-43 activates SHP-1 through direct interaction with SHP-1 proteins (Figure 3B). To validate the role of SHP-1 in mediating SC-43-induced apoptosis, we utilized PTPIII, a SHP-1 specific inhibitor. The protective effects of SC-43-induced apoptosis in HuCCT-1 and KKU-100 cells were noted after PTPIII administration (Figure 3C). After HuCCT-1 cells were transfected with SHP-1 siRNA, SC-43-induced apoptosis was reduced significantly in SHP-1-silencing HuCCT-1 cells, comparing to cells transfected with control si-RNA (Figure 3D, left). Then, we generated HuCCT-1 cells with constitutive, ectopic expression of myc-tagged SHP-1. SC-43 treatment in HuCCT-1 with high levels of SHP-1 showed more inhibition of p-STAT3 and more cell apoptosis, compared to cells transfected with empty vectors (Figure 3D, right). Taken together, these results suggested that SC-43 mediates the apoptotic effect in CCA cells through p-STAT3 inhibition by activating SHP-1 activity.

### SC-43 relieves autoinhibition of SHP-1 by interfering with the inhibitory N-SH2 domain

**Figure 4:**
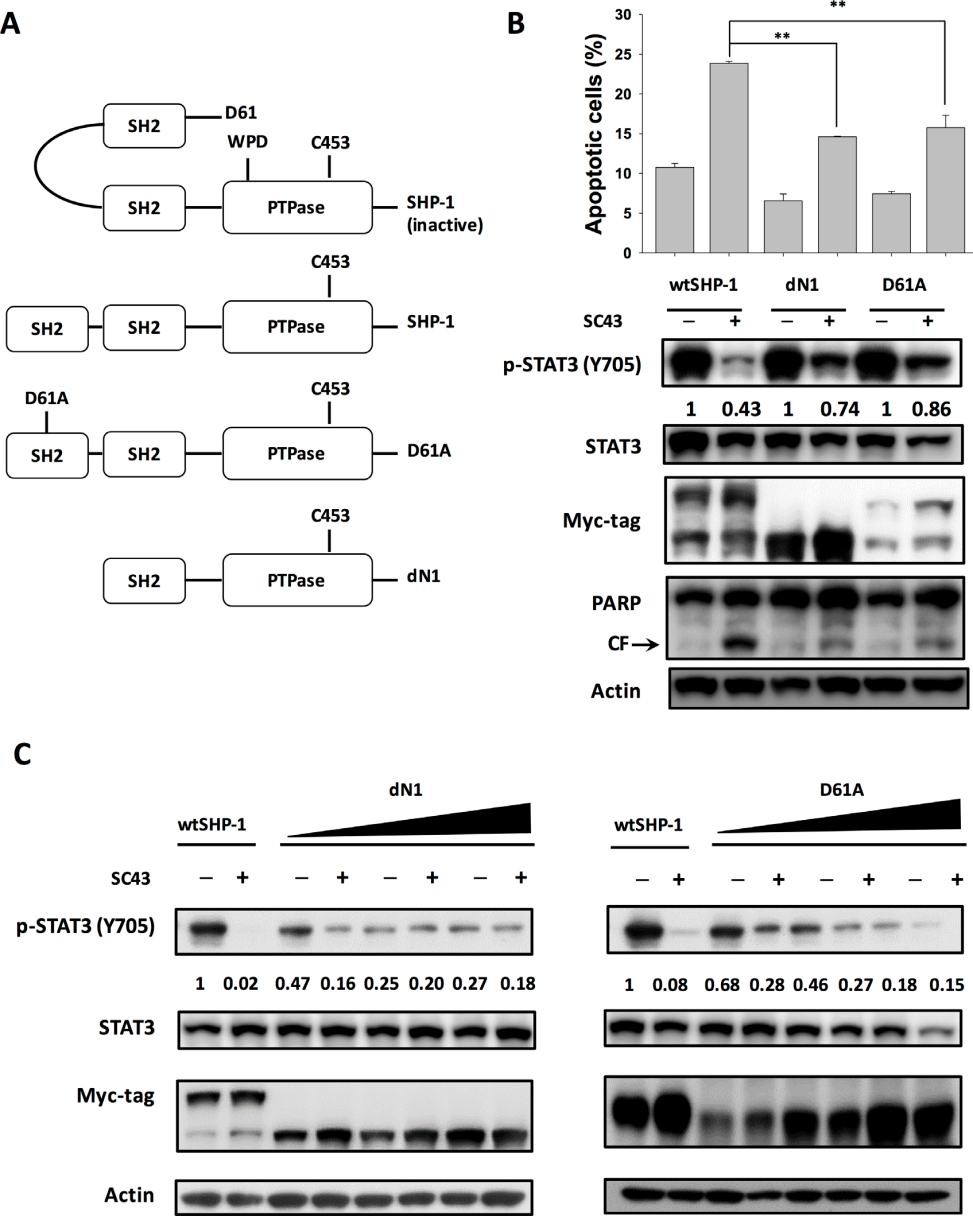
SC-43 activates SHP-1 by relieving the autoinhibition of the SH2 domain (**A**) schematic representation of deletion and single mutants of SHP-1. (**B**) dN1 and D61A impair SC-43-induced STAT3 signaling and apoptotic effect. Apoptotic assay was performed by sub-G1 analysis. Columns, mean; bars, SD (*n =* 3). **P < 0.01. (**C**) dose-dependent dN1 and D61A plasmid transfection suppressed p-STAT3 expression and decreased SC-43-induced p-STAT3 downregulation.

As the activity of SHP-1 was strongly regulated by the auto-inhibited 3D structure, we constructed wild-type, deletion of N-SH2 (dN1), and D61 single mutant (D61A) of SHP-1 to investigate the effect of SC-43 on different SHP-1 statuses (Figure 4A). The dN1 and D61A mutants resemble open (non-autoinhibition) forms. As demonstrated in Figure 4B, SC-43 induced significantly less p-STAT3 downregulation and apoptosis in HuCCT-1 cells expressing the dN1 and D61A mutants than in the wild-type control, suggesting that N-SH2 and PTPase catalytic domain are important for SC-43 induced effects. The conformational change of SHP-1 induced by dN1 and D61A counteracted the SC-43 effect. Furthermore, dose-escalation study of transfection of dN1 and D61A suppressed the expression of p-STAT3 and decreased SC-43-induced p-STAT3 inhibition (Figure 4C). These results indicated that relieved SHP-1 counteracts the SC-43-induced anti-CCA effect. SC-43, therefore, potentially relieves auto-inhibition of SHP-1, leading to p-STAT3 signal downregulation.

### Effect of SC-43 on CCA xenograft tumor growth *in vivo*

**Figure 5:**
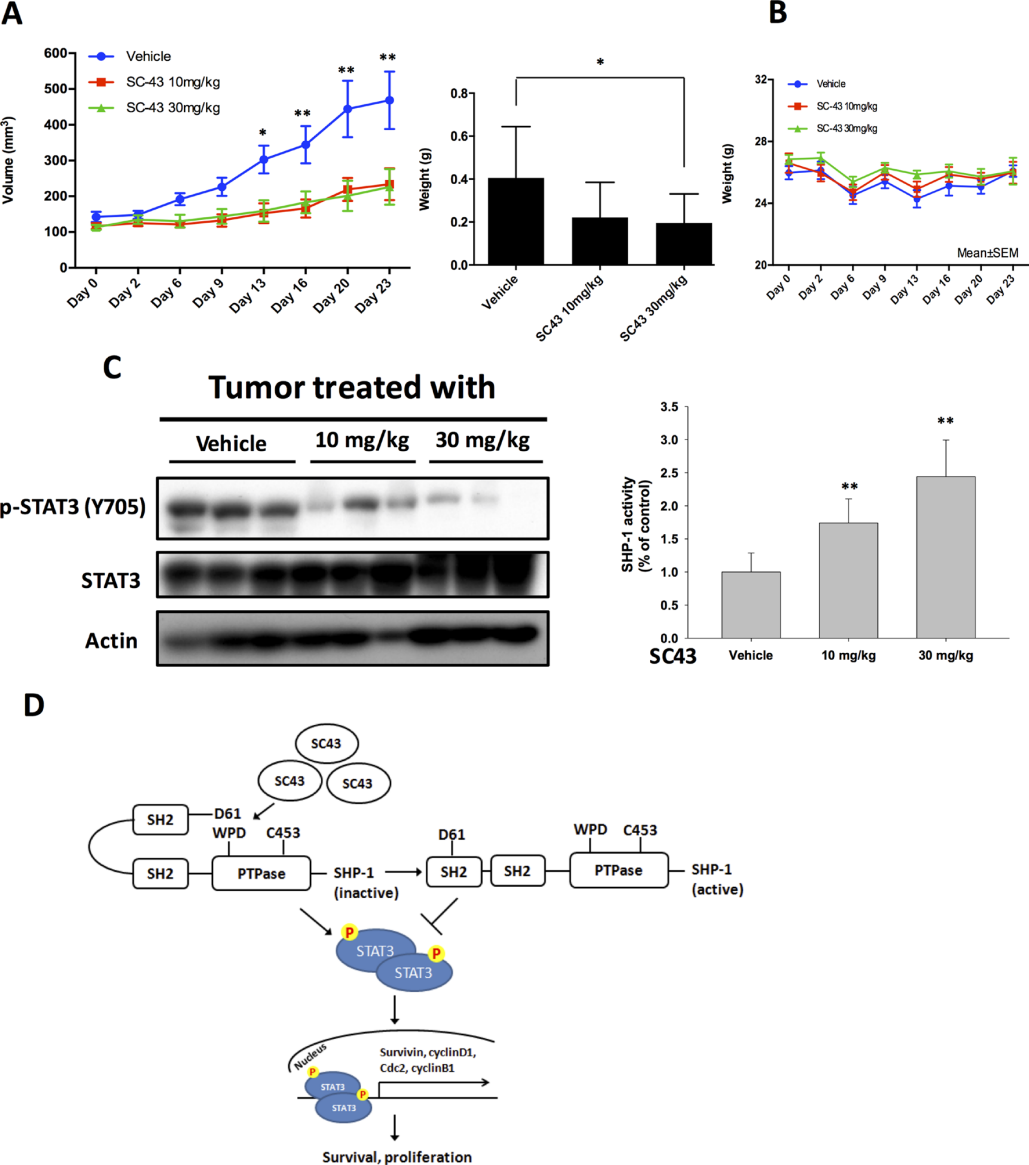
*In vivo* effects of SC-43 in CCA xenograft animal model (**A**) SC-43 exhibited significant tumor growth inhibition in a HuCCT1-bearing CCA subcutaneous model. Left, mice received SC-43 at 10 mg/kg/day or 30 mg/kg/day and tumor growth was measured twice weekly. Point, means; bars, SE (*n* ≥ 9). Right, tumor weight at the end of treatment. *P < 0.05; **P < 0.01. (**B**) Body weight of mice with SC-43 treatment. (**C**) left, analysis of p-STAT3 and STAT3 in HuCCT1 tumors. Right, SHP-1 phosphatase activity in HuCCT-1 tumors. Columns, mean; bars, SD (*n* ≥ 6). **P < 0.01. (**D**) summary model. SC-43 induced potent apoptosis in CCA by relieving the inhibitory N-SH2 domain of SHP-1 and downregulating p-STAT3, cyclin B1 and Cdc2.

In order to confirm that using SC-43 to inhibit p-STAT3 has potentially clinical relevant implications in CCA, we established a CCA xenograft model to evaluate the effect of SC-43 *in vivo*. In our study, SC-43 not only inhibited HuCCT-1 xenograft tumor size but also tumor weight significantly (Figure 5A). The side effect of SC-43 was well-tolerated and no observable signs of toxicity were noted. All the experimental animals had stable body weights throughout the whole treatment course (Figure 5B). The protein expression was checked to confirm the correlation between the biological response observed *in vivo* and the molecular mechanism discovered *in vitro*. The p-STAT3 level was reduced after SC-43 treatment. Moreover, SC-43 increased SHP-1 phosphatase activity compared with the control group (Figure 5C). A schema summarizing the molecular mechanism of SC-43 in sensitive CCA cells is presented in Figure 5D: SC-43 activates SHP-1 phosphatase by impairing the inhibitory N-SH2 domain, and contributes to p-STAT3, downstream cyclin B1 and Cdc2 downregulation, leading to G2-M arrest and cancer cell apoptosis.

## DISCUSSION

CCA is a fetal disease with dismal prognosis that lacks efficient medical management. There are few clinical trials incorporating molecularly targeted agents reported in this aggressive biliary tract malignancy. Therefore, novel treatment choice is of urgent need. In this article, we introduced a novel sorafenib derivative, SC-43, with potential therapeutic effects in CCA. We revealed that SC-43 demonstrates a significant anti-proliferative effect in CCA through p-STAT3 pathway inhibition. SHP-1 is a major determinant in the apoptosis effect of SC-43. By upregulating SHP-1, SC-43 induced SHP-1-dependent p-STAT3 downregulation. SC-43 directly interacts with SHP-1 SH2 domain and relieves its auto-inhibition structure. These findings not only increase current understanding of the SHP-1/STAT3 pathway but also support the rationale for targeting SHP-1 in the future development of therapies for CCA.

Chronic inflammation triggers cellular events that can promote malignant transformation of cells and carcinogenesis. Several inflammatory mediators, such as IL-6 and TGF-β, have been shown to participate in both the initiation and progression of cancer. STAT3 is crucial in initiating and maintaining a procarcinogenic inflammatory microenvironment, both at the beginning of malignant transformation and during further cancer progression [20–25]. IL-6/STAT3 remains a cornerstone factor regulating cholangiocyte growth and survival [26, 27]. There is a growing body of evidence showing that IL-6/STAT3 signaling is clinically significant in CCA. Banchob et al. revealed that elevated IL-6 level after liver fluke *O.viverrini* infection correlated with formation of advanced periductal fibrosis, suggesting the importance of IL-6 signaling in CCA formation [13]. The expression of IL6 is upregulated in cancer cells and serum in patients with CCA [28, 29]. Furthermore, expression of STAT3 is associated with poor histological differentiation and adverse prognosis in patients with cholangiocarcinoma [30]. In this study, we demonstrated that p-STAT3 is a major target of SC-43, which is a sorafenib derivative without kinase inhibitor activity in CCA cells. The apoptosis of CCA cells after SC-43 administration is correlated with p-STAT3 downregulation, suggesting that STAT3 is a potential target in treating CCA.

Src homology region 2 (SH2) domain-containing phosphatase 1, SHP-1, acts as a negative regulator of phosphorylated STAT3 (p-STAT3). SHP-1 is a nonreceptor protein tyrosine phosphatase (PTP), composed of two SH2 domains that bind phosphotyrosine, a catalytic PTP domain, and a C-terminal tail. The phosphatase activity of SHP-1 depends on the variation of its 3D structure, as evidenced by its open- or closed-form chemical structure. The N-SH2 domain protrudes into the catalytic domain to directly block the entrance into the active site, and the highly mobile C-SH2 domain is thought to function as an antenna to search for the phosphopeptide activator [31–33]. Previously we demonstrated that SC-43 activates SHP-1 through direct interaction with SHP-1 proteins in HCC cells [18], and this finding was also verified in breast cancer cells [34]. In the study on HCC cells, we provided a molecular docking model that sorafenib and SC-43 docked into the interface of N-SH2 and PTPase domain, we hypothesized that sorafenib and SC-43 could bind to the N-SH2 domain and subsequently releases and activates the PTPase domain. The interaction of sorafenib (or SC-43) and the N-SH2 domain might lead to a release of the D61 catalytic site and activation of SHP-1 activity [18]. In current study, the hypothesized mechanism of SC-43 was supported by using dN1 (deleted N-SH2) and D61A mutant SHP-1- overexpressing cells; SC-43 exerted less p-STAT3 downregulation and apoptosis-inducing effects in these mutant SHP-1 over-expressing cells, compared to wild-type SHP-1-expressing cells. These results confirmed that the SH2 domain is a critical docking site of SC-43. However, given the fact that ectopic expression of STAT3 (thus increased p-STAT3) did not completely rescued the SC-43 induced apoptosis (Figure 2C), and that SHP-1 inhibitor PTP3 partially reduced the effect of SC-43 (Figure 3C), it is possible that there may also be other pathways involved in the effect of SC-43 induced apoptosis. Since SC-43 increases SHP-1 activity, it is reasonable that SC-43 induced effects can be mediated by other SHP-1-dependent substrates. Alternatively, other SHP-1 independent pathways may also be involved in SC-43 induced effects. More studies are needed to elucidate whether other pathways are also involved in SC-43 induced effects in CCA cells.

It is clear that the cell-cycle checkpoints can regulate the quality and rate of cell division. Agents that can increase G2-M arrest have also been associated with enhanced apoptosis . For example, Jackson *et al.* [35] demonstrated that the Chk1 indolocarbazole inhibitor (SB-218078) enhanced G2-M arrest and cytotoxicity in HeLa cells. Hirose *et al.* [36]revealed that temozolamide induced G2-M arrest-related apoptosis in glioma cells. With regards to the higher G2-M arrest proportion, we hypothesized that SC-43 induced CCA cells apoptosis through G2-M arrest. In our study, levels of expression of the cyclin B1 and Cdk1 (Cdc2), the major regulatory protein and kinase for progression from G2 to M phase were markedly reduced after SC-43 treatment. It is known that STAT3 signaling axis induced cell proliferation through binding to cdc2 [23, 37, 38]. Furthermore, STAT3 promotes mitosis process primarily by stimulating transcription of cyclin B1 and other regulatory proteins, such as c-myc and cyclin D1 [39–43]. In our presenting study, SC-43 induces apoptosis with downregulation of STAT3 in sensitive CCA cells. In addition, SC-43 promotes G2-M arrest by reducing transcription level of cyclin B1 and Cdc2 protein.

SHP-1 is predominantly expressed in hematopoietic and epithelial cells. Notably, SHP-1 generally acts as a negative regulator in a variety of cellular signaling pathways [44, 45]. However, the underlying molecular mechanism by which SHP-1 is involved in carcinogenesis is not completely understood. Some studies have indicated that SHP-1 is a potential tumor suppressor gene in cancer formation [46–49]. However, the clinical value of cancer treatment by targeting SHP-1 is still under investigation. According to a few reports [50–52], several agents that regulate SHP-1 activity have been identified, and some have found efficacy against different cancer cells. In our previous work, we disclosed that regorafenib, a multiple protein kinase inhibitor, exerts anti-tumor effects by enhancing SHP-1 activity [50]. Dovitinib, another multiple protein kinase inhibitor, acts as a novel radiosensitizer in HCC by upregulating SHP-1 [51]. Moreover, SHP-1 activation by a novel Bcl-2 inhibitor derivative was found to induce HCC autophagy [52]. In the present study, we demonstrated that SC-43 has a SHP-1-dependent apoptotic effect in CCA cells. Therefore, our data strengthen the rationale for targeting the SHP-1/STAT3 pathway as a novel anti-cancer therapy. Taken together, these structurally unrelated agents show a common target in various cancer cells suggesting that SHP-1 may be a potential therapeutic target.

In this study, a total of 40 cases with pathologically confirmed CCA were analyzed in this study. About half (21 of 40, 52.5%) of the specimens revealed only weak positive p-STAT3 expression. We have added Supplementary Figure 2 showing some examples of weak-staining p-STAT3 in our CCA samples (Supplementary Figure 2) There are several possible reasons for the relatively low p-STAT3 expression in current study. First, the protein phosphorylation is usually dynamic and rapidly degraded. Second, the level of phosphorylated protein is relatively limited and difficultly detected. Third, the limitation of IHC for FFPE tissue due to the adverse influence of formalin upon antigenicity and the great variation in processing procedures. Fourth, in this retrospective study, some specimens have been preserved for more than ten years, therefore the quality of IHC in these FFPE tissue is inevitably diminished.

## MATERIALS AND METHODS

### Reagents and antibodies

SC-43 was dissolved in dimethyl sulfoxide and then added to the cells maintained in RPMI 1640 medium without FBS. SHP-1 inhibitor (PTPIII) was purchased from Calbiochem. Antibodies, such as cyclin D1, STAT3, phospho-STAT3 (Tyr705), survivin, and caspase-9 were purchased from Cell Signaling (Danvers, MA). Other antibodies, such as cyclin B1 and Cdc2 were purchased from Abcam (London).

### Cell culture and western blot analysis

The HuCCT-1 cell line was purchased from Riken BRC (Riken BioResource Center) (Saitama, Japan). The KKU-100 cell line was obtained from JCRB Cell Bank (Japanese Collection of Research Bioresources Cell Bank) (Osaka, Japan). The CGCCA cell line was kindly provided by Taipei Veterans General Hospital. HuCCT-1 cells was routinely cultured in RPMI 1640 (Invitrogen/Life Technologies, Saint Aubin, France), while KKU-100 and CGCCA cells were cultured in Dulbecco’s modified Eagle’s medium (Gibco/Life Technologies, Grand Island, NY, USA). All CCA cells were supplemented with 10% heat-inactivated fetal bovine serum (Gibco/Life Technologies, Grand Island, NY, USA), 100 μg/mL streptomycin sulfate, and 100 μg/mL penicillin, in a humidified atmosphere containing 5% CO_2_ at 37°C.

Lysates of CCA cells treated with drugs at the indicated concentrations for various periods of time were prepared for immunoblotting of p-STAT3, STAT3, etc.

Whole-cell lysates were resolved by sodium dodecyl sulfate polyacrylamide gel electrophoresis. Proteins were transferred onto a polyvinylidene difluoride membrane (Millipore, Billerica, MA, USA) and incubated with the primary antibody, and then incubated with horseradish peroxidase–conjugated secondary antibodies. Specific proteins were detected using enhanced chemiluminescence reagent.

### Cell viability and proliferation of CCA cell lines *in vitro*

Cell viability and proliferation of CCA cells treated with or without SC-43 were assessed by colorimetric assay using 3-(4,5-dimethylthiazol-2-yl)-2,5 diphenyltetrazolium bromide (MTT). Cells were plated in a 96-well plate in 100 μl DMEM per well and cultured for up to 72 hours. Cells were incubated for four hours at 37°C with MTT; after incubation, medium was removed and cells were treated with dimethyl sulfoxide (DMSO) for five minutes. Viability was evaluated by ultraviolet absorption spectrum at 540 nm with a Microplate Reader Model 550 (Bio-Rad, Richmond, CA, USA). Experiments were performed three times in duplicate.

### Apoptosis analysis

Drug-induced apoptotic cell death was assessed by measuring apoptotic cells by flow cytometry (sub-G1 analysis of *propidium iodide*-stained cells) and western blot analysis of caspase 3 cleavage.

### Generation of CCA cells with ectopically expressed STAT3

STAT3 cDNA (KIAA1524) were purchased from Addgene plasmid repository (http://www.addgene.org/). HuCCT-1 cells with ectopic expression of STAT3 derived from a single-stable clone were prepared for *in vitro* assay for STAT3 target validation. Briefly, following transfection as previously prescribed [19], HuCCT-1 was incubated in the presence of G418 (0.78 mg/ml). After 8 weeks of selection, surviving colonies, i.e., those arising from stably transfected cells, were selected and individually amplified. Control cells were transfected by empty vectors.

### Gene knockdown using siRNA

Smart-pool siRNAs, including control (D-001810–10), SHP-1 (PTPN6, L-009778- 00-0005), and STAT3 were all purchased from Dharmacon (Chicago, IL, USA). Plasmids of human wild-type STAT3 and SHP-1 (PTPN6) were encoded by pCMV6 vector with myc-tag. For mutant-type SHP-1 expression, we generated two plasmids, designated dN1 and D61A, with a truncated N-SH2/PTP domain and aspartic acid at 61 changed to an alanine residue, respectively. Both plasmids were cloned into pCMV6 entry vector. These plasmids or siRNAs were subsequently transfected into cells by using Lipofectamine 2000 Reagent (Invitrogen, CA, USA).

### SHP-1 phosphatase activity assay

After treatment of SC-43, the cellular protein extracts were incubated with anti-SHP-1 antibody in immunoprecipitation buffer (20 mM of Tris-HCl [pH 7.5], 150 mM of NaCl, 1 mM of ethylenediaminetetraacetic acid, 1% NP-40, and 1% sodium deoxycholate) overnight. Protein G-Sepharose 4 Fast flow (GE Healthcare Bio-Science, NJ, USA) was added to each sample, followed by incubation for 3 h at 4°C with rotation. A RediPlate 96 EnzChekR Tyrosine Phosphatase Assay Kit (R-22067) was used for SHP-1 activity assay (Molecular Probes, Invitrogen, CA, USA).

### Xenograft tumor growth

Male NCr athymic nude mice (5–7 weeks of age) were obtained from the National Laboratory Animal Center (Taipei, Taiwan, ROC). The mice were housed in groups and maintained in an SPF-environment. All experimental procedures using these mice were performed in accordance with protocols approved by the Institutional Animal Care and Use Committee of Cardinal Tien Hospital. Each mouse was inoculated orthotopically to the mouse mammary pads with 5 × 10^6^ HuCCT-1 cells suspended in 0.1 mL serum-free medium containing 50% Matrigel (BD Biosciences, Bedford, MA) under isoflurane anesthesia. Tumors were measured using calipers and their volumes calculated using a standard formula: width^2^ × length × 0.52. When tumors reached around 100 mm^3^, mice were administered orally with SC-43 at 10 mg/kg or 30 mg/kg daily by oral gavage. Controls received vehicle (1× phosphate buffered saline). Upon termination of treatment, mice were sacrificed and xenografted tumors were harvested and assayed for tumor weight, SHP-1 activity, and molecular events by western blot analysis.

### Immunohistochemistry

Immunohistochemical (IHC) stains were performed, using the Ventana BenchMark XT automated stainer (Ventana, Tucson, AZ, USA). Briefly speaking, 4-μm thick sections would be cut consecutively from formalin-fixed, paraffin-embedded (FFPE) human tissue. Sections would be mounted and allowed to dry overnight at 37°C. After deparaffinization and rehydration, slides would be incubated with 3% hydrogen peroxide solution for 5 minutes. After a washing procedure with the supplied buffer, tissue sections were repaired for 40 minutes with ethylenediamine tetra-acetic acid. The slides were then incubated with primary antibodies against p-STAT3 (1:50; Cell Signaling) for 60 minutes at 37°C and overnight at 4°C. Negative control slides processed without the primary antibody were included for each staining. After three rinses in buffer, the slides were incubated with the secondary antibodies (unbiotinylated antibody; EnVisionTM System; HRP, anti-mouse/rabbit, DakoCytomation; Dako, Glostrup, Denmark). Tissue staining were visualized with a 3,3′-diaminobenzidine (DAB) substrate chromogen solution (DakoCytomation). Slides were counterstained with hematoxylin, dehydrated, and mounted. Samples which express these markers strongly served as the positive controls. The mean percentage of positive tumor cells was determined in at least five areas at ×200 magnification. All slides were evaluated by experienced pathologists who reviewed the slides together and reached a consensus. Positive expression for p-STAT3 was defined as > 25% nuclear staining with greater than moderate staining intensity of tumor cells.

### Statistical analysis

All data are expressed as mean ± SD or SE. Statistical comparisons were based on nonparametric tests. *P value* less than 0.05 was defined as statistical significance. All statistical analyses were performed using SPSS for Windows software, version 19.0 (SPSS, Chicago, IL, USA).

## CONCLUSIONS

Novel sorafenib derivative, SC-43, exerts a G2-M arrest and anti-proliferative effect on CCA cells through a SHP-1-dependent STAT3 downregulation pathway by interrupting SHP-1 auto-inhibition. This study reinforced the hypothesis that targeting STAT3 in this inflammatory malignancy may be a useful therapeutic strategy, and provides an innovative approach to CCA management. Further study is warranted to explore the details of the SHP-1/STAT3 pathway in the development of disruptive molecular-targeted therapy for CCA.

## SUPPLEMENTARY MATERIALS FIGURES


