# Supplementary material for: Targeting SHP-1-STAT3 signaling: A promising therapeutic approach for the treatment of cholangiocarcinoma
Source: Oncotarget. 2017 May 10;8(39):65077–89. doi: 10.18632/oncotarget.17779 (PMC5630313; doi:10.18632/oncotarget.17779)
Supplement: Supplementary file 1 [file oncotarget-08-65077-s001.pdf]

Targeting SHP-1-STAT3 signaling: A promising therapeutic approach for the treatment of cholangiocarcinoma

SUPPLEMENTARY MATERIALS

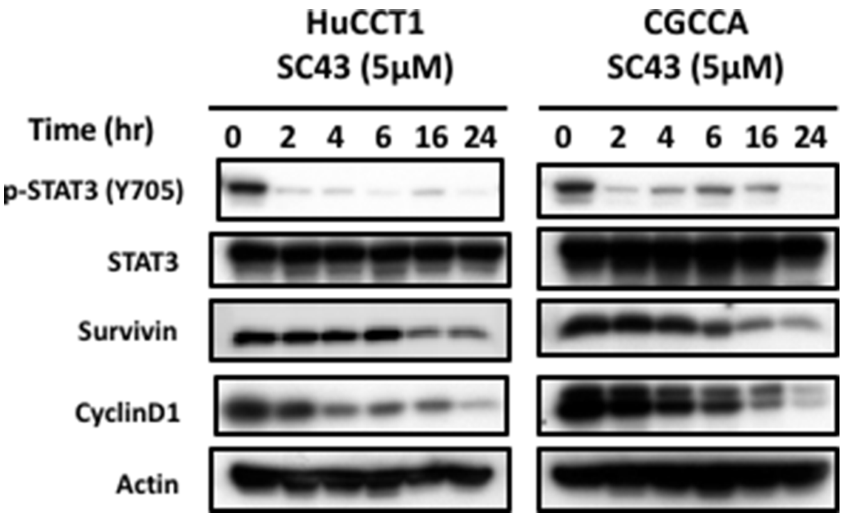

Supplementary Figure 1: SC-43 induced p-STAT3 inactivation in HuCCT-1 and CGCCA cells in a time-dependent manner.

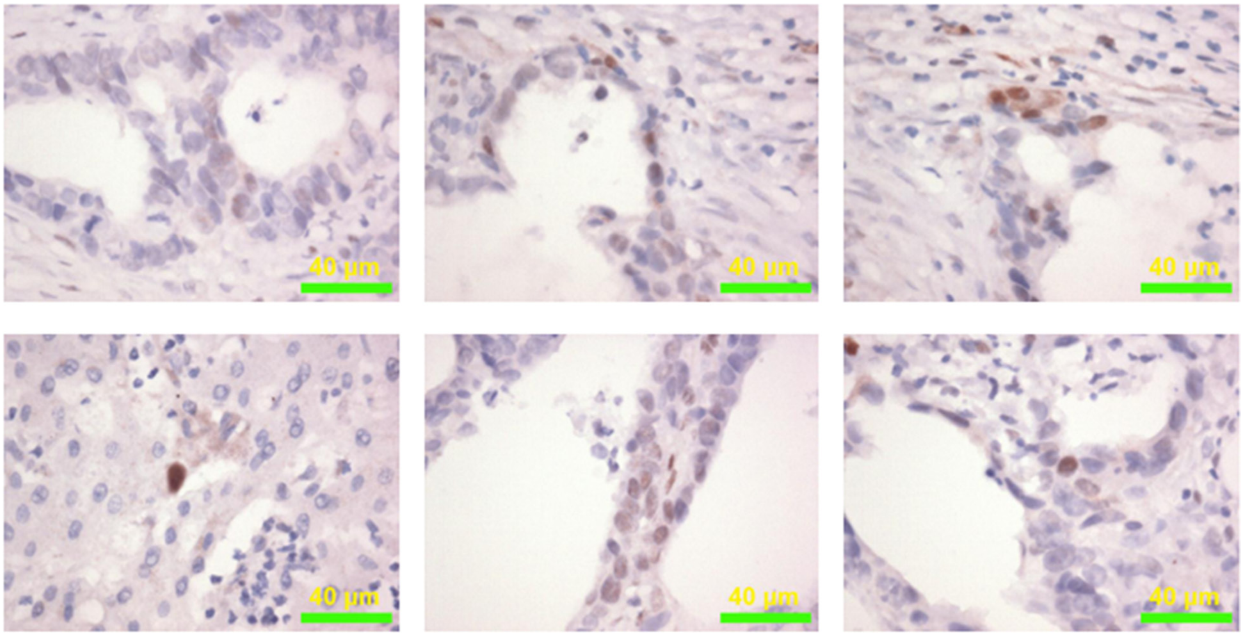

Supplementary Figure 2: Immunohistochemical (IHC) staining for p-STAT3 in CCA tumor. Examples of weak-staining p-STAT3 in our human CCA specimen.
